# How the Behavior Change Content of a Nationally Implemented Digital Diabetes Prevention Program Is Understood and Used by Participants: Qualitative Study of Fidelity of Receipt and Enactment

**DOI:** 10.2196/41214

**Published:** 2023-01-11

**Authors:** Lisa M Miles, Rhiannon E Hawkes, David P French

**Affiliations:** 1 Manchester Centre for Health Psychology Division of Psychology and Mental Health University of Manchester Manchester United Kingdom

**Keywords:** diabetes prevention, digital interventions, behavior change, fidelity, receipt, enactment, mobile phone

## Abstract

**Background:**

The National Health Service Digital Diabetes Prevention Programme (NHS-DDPP) is a program for adults in England at risk of developing type 2 diabetes mellitus (T2DM). It is based on NHS England specifications that stipulate specific behavior change techniques (BCTs), that is, *active ingredients* to produce behavior change to target diet and physical activity. Now rolled out nationally, the NHS-DDPP is being delivered by 4 independent providers as a 9-month intervention via apps, educational material, and remote health coaching. To optimize effectiveness, participants need to be able to understand and use behavior change content (eg, goal setting and problem solving) of an intervention delivered to them digitally. Previous research has shown that people benefit from support to aid the understanding and use of BCTs.

**Objective:**

The objectives of this qualitative study were to evaluate how participants in the NHS-DDPP understand and use BCT content, investigate how participants describe the role of health coaches in supporting their behavior change, and examine how the understanding and use of behavior change content of the NHS-DDPP varies across providers.

**Methods:**

In total, 45 service users were interviewed twice by telephone at 2 to 4 months into, and at the end of, the program. Topics included participants’ understanding and use of key BCTs to support self-regulation (eg, goal setting) and the support they received via the program. Transcripts were analyzed thematically, informed by the framework method.

**Results:**

Participants described their understanding and use of *some* behavior change content of the program as straightforward: use of BCTs (eg, self-monitoring of behavior) delivered digitally via provider apps. Participants valued the role of health coaches in supporting their behavior change through the emotional support they offered and their direct role in delivery and application of some BCTs (eg, problem solving) to their specific circumstances. Participants expressed frustration over the lack of monitoring or feedback regarding their T2DM risk within the program. Variations in the understanding and use of behavior change content of the NHS-DDPP were present across provider programs.

**Conclusions:**

Health coaches’ support in delivery of key components of the program seems to be pivotal. To improve the understanding and use of BCTs in digital interventions, it is important to consider routes of delivery that offer additional interactive human support. Understanding of some self-regulatory BCTs may benefit from this support more than others; thus, identifying the optimal *mode of delivery* for behavior change content is a priority for future research. The NHS-DDPP could be improved by explicitly setting out the need for health coaches to support understanding of some self-regulatory BCT content such as *problem solving* in the service specification and amending the discharge process so that knowledge of any change in T2DM risk is available to participants.

## Introduction

### Background

More than 420 million people are living with diabetes worldwide [1], and this number is projected to increase by 25% in 2030 and by 51% in 2045 [2]. Worldwide, diabetes-related health expenditure was estimated to be US $760 billion in 2019, and this is expected to grow to US $825 billion per year by 2030 [3].

Type 2 diabetes mellitus (T2DM) is largely preventable through weight loss and improved diet and physical activity; therefore, several diabetes prevention programs have been implemented around the world [4,5]. These programs have traditionally been developed as in-person group-based behavior change programs, which have demonstrated effectiveness in terms of preventing progression to T2DM and reducing body weight and blood glucose levels [6]. These programs include the Healthier You National Health Service Diabetes Prevention Programme (NHS-DPP) for adults in England, which was launched in 2016 [7,8]. Nevertheless, the in-person and group-based program formats are not suitable for everyone; barriers to participation include work and caring commitments as well as transportation [9,10].

Digital health interventions are widely viewed as having advantages in terms of cost-effectiveness, scalability, and personalization [11]. Systematic reviews have shown digital diabetes prevention interventions to be effective for weight loss [12,13] and improvements in glycemic control in patients with prediabetes [12]. In an uncontrolled pilot study of a digital version of the NHS-DPP, participation in the program was associated with clinically significant reductions in weight (–3.1 kg) and glycated hemoglobin (HbA1c) levels (–1.6 mmol/mol) at 12 months [14]. After this successful pilot, the NHS-DPP was extended in 2019 to also include a digital version of the program, the NHS Digital Diabetes Prevention Programme (NHS-DDPP).

The NHS-DDPP is currently delivered by 4 independent providers commissioned to deliver the 9-month behavior change program, based on an NHS England service specification [15], which itself was based on evidence outlined in a systematic review [6] and a National Institute of Health and Care Excellence public health guideline [16]. The 4 providers use a range of digital features to deliver program content to participants (Table 1), largely relying on smartphone apps that include functions for self-monitoring behaviors (eg, steps) and outcomes (eg, weight) as well as access to other resources; educational articles on T2DM risk, diet, and physical activity; and health coaching, which usually involves regular contact between a health coach and an NHS-DDPP participant via telephone, video call or web-based chat. Although broadly similar approaches to delivery of content are used across providers, there are some important differences, including the frequency and format of communications with health coaches (refer to *Core phase of program* in Table 1).

**Table 1:** Digital features of the National Health Service Digital Diabetes Prevention Programme delivered by providers.

	Provider A	Provider B	Provider C	Provider D
Materials provided to service user (for use 1-40 weeks)	Program app	Program app and program handbook	Program app	Program app, program handbook, recipe book, wireless scales, and activity tracker
Core phase of program (1-12 weeks)	Health coaching via series of scheduled telephone calls and web-based chat; 42 web-based articles	Access to health coaches via chat function; weekly web-based articles; optional web-based discussion forum	Health coaching via initial telephone call, then regular video messages and web-based chat; health coach sends documents and videos to service users; optional web-based discussion forum	Health coaching in a WhatsApp group of approximately 10 people (access to health coach in group or one-on-one chat); web-based articles unlocked daily
Maintenance phase of program (13-40 weeks)	Health coaching via series of scheduled telephone calls and web-based chat; 42 web-based articles	Access to health coaches via chat function; weekly web-based articles; optional web-based discussion forum	Health coaching via video calls and web-based chat; health coach sends documents and videos to service users; optional web-based discussion forum	Eight optional 4-week web-based courses; optional web-based discussion forum

A significant feature of the NHS service specification is that it specifies 19 behavior change techniques (BCTs) that should be delivered within the NHS-DDPP [15]. BCTs are observable, irreducible, and replicable components of an intervention, often referred to as the *active ingredients* designed to change behavior (eg, goal setting, problem solving, and feedback) [17]. A number of these BCTs address self-regulatory processes, that is, those in a feedback loop consisting of goal setting, recognizing inconsistencies between goals and current behavior, and developing plans to reduce these inconsistencies [18]. Their presence in the NHS service specification is a result of existing evidence that suggests that these self-regulatory BCTs are an important component of behavioral interventions relevant to T2DM prevention [6,16].

To fully understand why interventions are ineffective or effective we need to understand whether these interventions are designed, delivered, and received as planned (fidelity). Without an assessment of fidelity, the reported effectiveness of an intervention could be a function of either an effective intervention or the influence of other unknown factors added to, or omitted from, the intervention [19]. Findings from fidelity assessments can often inform implementation of programs in practice and identify solutions for more effective rollout of programs [20].

Five domains of fidelity (design, delivery, training, receipt, and enactment) have been conceptualized by the National Institutes of Health Behavior Change Consortium (NIH-BCC) [21]. Our work on the design of the NHS-DDPP indicates that fidelity to the evidence base is better than that previously documented for the face-to-face program [22]. This study focuses on receipt and enactment, which have been less well studied than other domains of fidelity [23-25].

In our previous study of receipt of the face-to-face NHS-DPP [26], participants’ understanding of the self-regulatory BCT content of the NHS-DPP was mixed and varied by BCT. Although some BCT content such as *self-monitoring of behaviors* was understood with ease, participants struggled to recall *action planning* or *problem solving* or found these techniques challenging to understand unless additional support was provided. The need for support to understand some BCTs suggests that *how* a BCT is delivered is likely to have an impact on how it is received and used by the target population.

One model for providing *support* alongside behavior change content is through health coaching; this is a key mode of delivery in the NHS-DDPP. A key element of health coaching is providing person-centered support [27,28], and it is usually provided in the context of a consistent ongoing relationship with a human coach who is trained in specific behavior change, communication, and motivational skills [28]. On the basis of this work, any impact of health coaching as a mode of delivery on the understanding and use of BCTs warrants further exploration.

With the increasing focus on developing digital behavioral interventions, it is important to understand how participants understand and use digitally delivered BCTs. End users of such interventions are not simply passive recipients but need to develop behavioral skills and cognitive strategies so that they can fully enact the key components of the intervention in their day-to-day lives in order to change their behaviors and thus prevent ill health [29]. Importantly, even if the program was designed and delivered with high fidelity, the NHS-DDPP might still not be as effective as it could be if people do not fully understand and use BCTs as intended.

On the basis of our previous findings [26] and calls for more qualitative work on receipt and enactment [29], the research team decided to assess fidelity of both receipt and enactment qualitatively. We previously found that participants tend to talk naturally about receipt and enactment of BCTs together in interviews. Using qualitative methods to investigate receipt and enactment also allows exploration of *how* participants understand and use key components of an intervention, including their description of the skills they have learned (cognitive and behavioral), what support they needed to understand and use them, and their confidence in implementing them.

### Objectives

To date, participants’ understanding and use of BCTs delivered in the nationally implemented NHS-DDPP is unknown, as is the need for support to help them understand these BCTs. We have taken an approach that aimed to describe *how* program participants understand and use BCTs, in line with *receipt* and *enactment* as defined by the NIH-BCC [21], rather than categorize understanding as correct or incorrect. The primary objective of this study was to evaluate how participants in the NHS-DDPP understand (receipt) and use (enactment) the BCT content of the program. Second, based on our previous findings that showed the importance of support for helping participants to understand BCTs, we investigated how participants describe the role of health coaches in supporting their change in behaviors. Third, we examined how the understanding and use of behavior change content of the NHS-DDPP varies across providers.

## Methods

### Participants

Participants were people who had participated in the NHS-DDPP and were invited to take part in this study between February 2021 and April 2021. Eligible participants were those who were 2 months into the program. Each provider was asked to send out invitations (by email along with a participant information sheet) to participants meeting these criteria over a period of 1 month. The demographic characteristics of people who initially responded and took part in an interview were reviewed using the demographic questionnaires completed at the time of the interview. Purposive sampling of the remaining respondents to the invitation was conducted with the aim of securing a broad representation of participants across providers, age, sex, and ethnic groups. Follow-up emails were sent by most (3/4, 75%) of the providers to target participants from deprived areas and minority ethnic groups (Multimedia Appendix 1). Participant postcodes were collected to allow calculation of the Index of Multiple Deprivation [30].

### Design and Procedure

Participants were interviewed twice: at 2 to 4 months into the program and then at 8 to 10 months as they came to the end of the program. The 2 time periods were selected so that participants could reflect on their experiences of the core part of the program (usually the first 12 weeks) and then at the end of the program to reduce the limitations of recall and to explore changes in the understanding and use of behavior change content as they progressed through the 9-month program.

After receiving invitations from the providers, potential participants contacted the research team to arrange an interview. Two researchers (LMM and REH) obtained participant consent by telephone (the conversation was recorded) and answered any questions before commencing the interviews. Interviews were conducted by telephone by the same 2 researchers and lasted between 30 and 60 minutes. Each interview was recorded and transcribed verbatim for analysis.

### Topic Guides

The topic guides (Multimedia Appendix 2) for the semistructured interviews focused on participants’ general experiences and engagement with the NHS-DDPP as well as their understanding and use of a range of self-regulatory BCTs (those where evidence supports their inclusion in a behavioral intervention): action planning, goal setting (for behaviors and outcomes), feedback (on behaviors and outcomes), self-monitoring (of behaviors and outcomes), and problem solving (refer to the study by Michie et al [17] for definitions). The topic guides focused on the understanding and use of these BCTs (addressing the receipt and enactment domains of fidelity) and were adapted from one used in a previous study [31] to allow participants to describe their understanding of the BCTs within the context of digital delivery (eg, use of apps and remote support from a health coach).

### Analysis

As each participant was interviewed twice, this qualitative analysis included a longitudinal element. Each participant’s 2 interviews (time points 1 and 2) were analyzed as a single unit to reflect service users’ descriptions of their behavior change journey as they progressed through the program rather than comparing findings at 2 time points. Our analytical approach was to consider the in-depth participant descriptions afforded by qualitative methods to describe *how* participants understand and use BCTs. Analysts were trained in the BCT taxonomy (version 1) [17]; therefore, they could interpret the understanding and use of specific BCTs from participants’ descriptions. Analysis was conducted in stages, using NVivo software (version 12; QSR International) to facilitate coding and analysis:

1. Time point 1 interviews were analyzed thematically. After initial familiarization with the transcripts, 2 researchers (LMM and REH) inductively coded 4 transcripts, which were then discussed in detail by all authors to agree upon the subsequent approach to coding. A decision was made to continue to inductively code the remaining transcripts, closely referring to the research questions. The remainder of the time point 1 interviews were then coded by LMM and REH working to an agreed coding framework of broad categories under which new inductive codes were added. This coding framework was discussed and refined throughout the process.
2. All authors met to discuss time point 1 interview coding and initial thoughts on candidate themes before analysis of the time point 2 interviews. A decision was made to code time point 2 interviews deductively, with reference to the refined coding framework (conducted by LMM and REH). Entirely new concepts were not identified in the time point 2 interviews; rather, there was further development of the concepts identified in the time point 1 interviews.
3. Once all coding was completed, all authors met to discuss and refine themes based on interview data from participants across all providers.
4. Framework matrices [32] were developed specifically to address the third research objective concerning variations across providers. Such charting allowed patterns of differences in findings across providers to be identified.
5. Final theme descriptions, including reference to the most pertinent provider differences, were then discussed and refined by all authors.

The approach we have taken to investigating understanding a BCT is in line with the definition of receipt in the NIH-BCC fidelity framework (12). We used the in-depth participant descriptions afforded by qualitative methods to describe *how* a participant understands BCTs and *how* support might aid their understanding of a BCT, rather than categorize their understanding as correct or incorrect, present or absent, or good or poor. Key features of understanding that we considered included (but were not limited to) participants’ recall of the technique within the program, their description of knowledge and performance of cognitive and behavioral skills, their confidence in implementing a technique, factors that enabled their use of a technique, and direct use of a technique as a strategy to change behavior.

### Ethics Approval

The wider program of research of which this study is a part was reviewed and approved by the North West Greater Manchester East NHS Research Ethics Committee (17/NW/0426; August 1, 2017). Full verbal consent was obtained from all participants included in this study.

## Results

### Demographic Characteristics

Of the 45 interviewees, 24 (53%) were women, and in terms of ethnicity, 10 (22%) identified as Asian, Black, or White Other. The participants’ median age was 59 (range 21-78) years. The majority (31/45, 69%) of the participants were from deciles 6-10 of deprivation (that is, areas of low deprivation; Table 2). Of the 45 participants, 36 (80%) were interviewed at time point 2. Refer to Multimedia Appendix 1 for details of participant recruitment.

Three themes were identified in the analysis:

- Importance of person-centered support
- Understanding and enactment of some BCT content without coaching
- Desire to know impact on T2DM risk

When present, any variation in the understanding and use of behavior change content of the NHS-DDPP across providers is articulated in the theme descriptions.

**Table 2:** Demographic characteristics of interview participants (N=45).

Characteristics		Values
**Provider of program that participants took part in, n (%)**		
	Provider A	12 (27)
	Provider B	11 (24)
	Provider C	10 (22)
	Provider D	12 (27)
Age (years), median (range)		59 (21-78)
**Sex, n (%)**		
	Female	24 (53)
	Male	21 (47)
**Ethnicity, n (%)**		
	White British	35 (78)
	Asian	5 (11)
	White Other	3 (7)
	Black	2 (4)
**Index of Multiple Deprivation, n (%)**		
	Decile 1 (most deprived)	4 (9)
	Decile 2	1 (2)
	Decile 3	2 (4)
	Decile 4	3 (7)
	Decile 5	4 (9)
	Decile 6	7 (16)
	Decile 7	3 (7)
	Decile 8	8 (18)
	Decile 9	5 (11)
	Decile 10 (least deprived)	8 (18)

### Theme 1: Importance of Person-Centered Support

#### Overview

Overall, the interviews clearly revealed how participants valued the role of health coaches in supporting their behavior change journey. However, for participants from provider B’s program, coaching input was notable by its absence (refer to Table 1 for variations in the format of health coaching across providers); for example, a participant from provider B’s program described a lack of personal support on the program:

Somebody contacted me to say you’ve been referred and gave me the information and gave me the password, emailed me some stuff, and then I just went on. I don’t think, recalling it, I’ve had any other contact with them...I think she did say somebody would ring me up every so often, but I haven’t heard from anybody.

Female aged 67 years, provider B

Participants from the other 3 providers described the value of health coaches in terms of their general (including emotional) support (refer to the Subtheme 1.1: Emotional Support Gained Through Coaching Relationship section) and also their direct role in delivery of, and supporting use of, BCTs (refer to the Subtheme 1.2: Coaching Instrumental in Delivery of Some BCTs section).

#### Subtheme 1.1: Emotional Support Gained Through Coaching Relationship

Participants clearly valued how health coaches could provide affirmation or positive reinforcement to support them in their behavior change journey; they described this as encouraging and motivating. In some cases, participants referred to the expertise or knowledge held by the health coach and felt that checking in with such an *expert* helped them to stay on track:

Well, because it motivates you and makes you feel proud that you’re obviously on the right track. And someone else who’s, you know, got skills and knowledge in that area also thinks you’re on the right track. So it’s kind of—well, it’s good.

Male aged 46 years, provider C

Several participants described a sense of accountability toward their health coach, largely because this person regularly checks in with the participant on their progress and reviews tracked information input into the app. In turn, participants described how this sense of accountability often affected their motivation:

Oh, because you’re constantly getting that affirmation, it’s constantly having that contact with someone that knows what they’re talking about, someone that has this as a career, and the importance of it, so it’s having that conversation with someone else, that encouragement, and having those goals, it’s like constant feedback, which is really, really important, I think.

Female aged 43 years, provider A

The fact that the program provides *someone there* who gives the participant time and attention—someone to ask questions of and talk through problems with—is important. Very often, participants described health coaches as responsive to their needs; they found it helpful to reach out to the health coaches to answer their specific questions. Overall, this suggests that interactive, person-centered support for behavior change is very much valued by service users:

I think it’s crucial to have a personal person, not just a computer program or an app. The fact there’s a human being at the end reviewing it and giving you feedback. I think it’s that accountability thing. I think that’s the main success of the program is having a human being there that you can ask for advice and support, and is monitoring, you know, the things that you input. Yeah, I think that’s the key to it.

Female aged 51 years, provider C

Furthermore, participants were very positive about the coaching *tone* of the relationship with their health coach. They frequently described their coaches as encouraging and nonjudgmental and enjoyed the praise, balanced feedback, and reassurance that was offered in their communications with them:

She’s on side with you rather than, um, being...putting judgment on you.

Female aged 53 years, provider D

#### Subtheme 1.2: Coaching Is Instrumental in Delivery of Some BCTs

Participants described how health coaches were involved directly in coaching them through a range of helpful techniques. One such technique is *problem solving*, where the discussion with the coach is an important part of identifying barriers and exploring options for how to overcome them:

Well, it does work because, you know, if I didn’t have someone to discuss it with, if I didn’t have these conversations I probably would have just kind of, well, I don’t know what the answer is, just given up, looked for sort of other options which wouldn’t have been as advantageous...I mean that was the most helpful thing for me actually, having someone that does this for a living, so knows the other options, knows the other avenues to explore.

Female aged 43 years, provider A

As a result, several participants were able to provide examples of problems they had solved (enactment) or at least a change in mindset that helped them to handle barriers that they encountered from time to time.

Health coaches also supported participants to set and review goals (particularly for behaviors more than outcomes), leading to an understanding of how goal setting can be implemented to facilitate behavior change. Conversely, participants undertaking provider B’s program tended to not recall being asked to set any goals via the NHS-DDPP; however, some were able to set themselves goals for outcomes (usually weight loss) without requiring additional support. This suggests that service users’ understanding of the BCT concerned with goal-setting for outcomes may be less reliant on coaching support.

The participants’ understanding of how goal setting could help them was similar for both behavioral and outcome goals. Notably, only a few participants recalled or accessed the related BCT *action planning* within the program (Multimedia Appendix 3). In some instances, the goal setting (behaviors) was also seen as a route by which to develop healthy routines and habits that could be maintained in the long term. Some participants also understood the value of discussing, reviewing, and changing goals with their health coach as they progressed through their behavior change journey:

I think working with the coach to set ones [goals] that are achievable and doing it gradually, and then upping them, it’s quite good because you can at least see that you’ve done it and then you can agree together to put them up. I think the big thing is them saying, you know, let’s make sure we’ve got something realistic here.

Female aged 55 years, provider C

A number of the participants also described how the discussions with the health coach had led to specific suggestions for them to implement new techniques according to their needs, such as food swaps (akin to the BCT *behavioral substitution*) or stress management (akin to the BCT *reduce negative emotions*). Furthermore, participants commonly described receiving very specific feedback on the dietary and physical activity information they had tracked as part of the program (akin to the BCT *feedback on behavior*):

It seems clear that he’s obviously analyzing and looking at what you’ve done, and analyzing that, ’cos he’ll offer specific feedback: “It’s great to see you’ve done a bit of running or you’ve been out on the bike.”

Male aged 48 years, provider C

Participants understood and were using such feedback, and they valued how this feedback was specific to them as individuals. The way in which coaches could personalize the program appropriately was described by some as a key feature of the program:

I think the coach thing is really invaluable, and the personalization, which I’ve already said. I think that’s what makes the difference. It’s not just education. It’s actually helping you apply it to yourself.

Female aged 55 years, provider C

### Theme 2: Understanding and Enactment of Some BCT Content Without Coaching

However, some BCT content in the program seemed to be straightforward for service users to understand and use without coaching support; for example, app functions for tracking behaviors and outcomes were a major feature of the provider apps, and participants could speak at length about whether and how these functions had been used.

The majority of the participants across all providers were self-monitoring some behaviors, particularly physical activity, followed by diet, and sometimes this continued to the time point 2 interviews. Participants were describing their use of the functions in the provider apps and seemed to be able to use self-monitoring techniques without much support. There was some variation in how participants described the ease or burden of self-monitoring behaviors, which may be related to the variations in self-monitoring methods used by different providers (such as manually logging, scanning, or taking photographs of meals), and in some cases, there was reference to other apps or tracking devices not directly provided within the NHS-DDPP, such as smartwatches that self-monitor physical activity.

The ways in which participants understood how self-monitoring behaviors helped to facilitate behavior change were consistent across providers; for example, reflecting on daily behaviors and making adjustments going forward (self-regulation); monitoring progress, particularly against goals set; and improved awareness of current behaviors. Some participants also enjoyed a sense of competition to either beat their own past scores or those of other participants in the program. Overall, this suggested that the BCT *self-monitoring of behaviors* seemed to be understood and used with ease without coaching support:

The odd time I will have a glass of red wine and then I know I’ve got to cut down on something else but, on the whole, I think it balances out quite well.

Female aged 75 years, provider B

Well, you just see what you’re doing. And, you know, you just think, oh, look, I haven’t really walked that much this week. I really need to do something about it. And get on with it.

Male aged 64 years, provider D

The findings described here regarding self-monitoring of behaviors were generally consistent across providers. However, there was some variation in how participants received feedback on behaviors, which seemed to be linked to whether such feedback was delivered by health coaches or automated via app functions. For providers A and C, participants mostly talked positively about such feedback delivered by health coaches (refer to the Subtheme 1.2: Coaching Instrumental in Delivery of Some BCTs section). For provider D, participants reported mixed responses on the automated feedback they received (informative or encouraging or unhelpful), whereas participants from provider B’s program described using a helpful feedback app feature to help them make healthier dietary choices.

The majority of service users, from all provider programs, were self-monitoring their body weight; this often continued up to the second interview. The service users clearly valued focusing on their outcomes. Participants talked about this technique (self-monitoring behavioral outcomes) providing a sense of focus or motivation. They liked to see the progress they had made, usually received as visual feedback on changes to body weight available through the provider apps, and felt good when weight loss had been achieved. They also self-monitored weight to regulate their behavior, that is, they made additional efforts to change behavior if they detected an increase in body weight:

It works, it is the key driver that keeps me in check. If my weight goes up on three or four consecutive days, which is rare, I will be saying, well, something is not working. You’ve either slipped into a bad habit, or, you know, you’re not taking it as seriously as you were...So it becomes a visual reminder as to where I am on this journey.

Male aged 57 years, provider D

Overall, understanding of the BCTs *self-monitoring outcomes* and *feedback on outcomes* (body weight) was evident in the interviews across participants taking part in all provider programs, and they could use these techniques effectively without additional coaching support.

### Theme 3: Desire to Know the Impact on T2DM Risk

At the second interview, participants often reflected on their perceptions of their overall success in terms of outcomes. As described earlier, participants were well informed about their progress on weight change. However, across many time point 2 interviews, participants expressed a general sense of frustration over the lack of monitoring or feedback regarding their T2DM risk (blood glucose and HbA1c levels) within the program. This was a consistent finding across participants from all provider programs. For some participants, by chance, their general practitioner had measured a follow-up T2DM risk (blood glucose and HbA1c levels), perhaps because of blood tests conducted for other health reasons; for these people, any beneficial impact on risk was positively received and important for them. However, for many, the route to be tested for T2DM risk within or via the NHS-DDPP was unclear, and they were left wondering about the impact of the program on their T2DM risk:

You know, no one’s actually looking out for me. It’s got to be me, right? I have to go and say, “Do you think you can take my blood sugar?”

Female aged 70 years, provider C

Their desire to understand their follow-up T2DM risk was partly related to a need to validate their efforts. Beyond any changes in body weight, participants wanted to know whether the program had worked for them:

I would personally benefit, I think, certainly back to the GP [general practitioner], take the same test and then somebody say, well, you were forty-two, you’re now twenty-six...That would round it off for me because I don’t know whether my diabetes stats are any lower, or any higher, even...So I think as a round up that would be useful for me because it validates the effort and the program and the...it would give me validation for it.

Male aged 57 years, provider D

## Discussion

### Principal Findings

NHS-DDPP participants described their understanding and use of *some* behavior change content of the program as straightforward; for example, use of BCTs delivered digitally via provider apps. Participants valued the role of health coaches in supporting their behavior change journey in terms of both the emotional support they offered and their direct role in delivery and application of BCTs to the participants’ specific circumstances. The findings regarding the role of health coaches do not reflect the experiences of participants from provider B’s program because this provider’s health coaching service is not proactively delivered to all participants. The understanding and use of the BCTs *problem solving* and *setting and reviewing goals* (particularly for behaviors) was aided by discussions with a health coach. Participants valued how the coaches’ feedback on behaviors was specific to them as individuals. Conversely, the BCTs *self-monitoring behaviors and outcomes* and *feedback on outcomes* seemed to be straightforward for service users to understand and use without coaching support. Participants expressed a general sense of frustration over the lack of monitoring or feedback regarding their T2DM risk within the program, which was partly related to a need to validate their efforts.

### Strengths and Limitations

The qualitative methodology for this study of fidelity is a particular strength, which has been called for by previous researchers [24,29]. As the majority (36/45, 80%) of the participants were interviewed twice, they had 2 opportunities to recall and describe their understanding and use of BCTs throughout the program; therefore, the risk of poor recall was minimized. Furthermore, our sample of participants (n=45) was diverse in terms of demographic characteristics. This sample size allowed us to elucidate key differences in the understanding and use of BCTs across providers. To date, the distinction between receipt and enactment as different domains tends to assume that the intervention takes place (during which participants understand content and gain skills), and then participants enact these skills and implement behavior change strategies in real life at a later point in time. In line with our previous recommendations [26], our approach of considering receipt and enactment together takes into account the 9-month duration of the program during which participants are expected to both understand *and* use the BCTs, that is, receipt and enactment are taking place concurrently.

Furthermore, this study provides a fidelity examination of a nationally implemented digital behavior change program where the research team members were independent of those who developed the NHS-DDPP. In particular, the ability to examine the fidelity of 4 separate providers delivering the same program and highlight important differences is unique. Such examinations of intervention fidelity are rare.

Although efforts were made to secure a broad representation of participants across age, sex, and ethnic groups, it is possible that those who proactively chose to take part in the study may be more likely to have had a positive experience of the program and are not representative of all participants in the NHS-DDPP. The age and sex characteristics of our participants were similar to those of participants of the pilot NHS-DDPP [14], but our sample was characterized by lower levels of deprivation and less ethnic diversity compared with the sample of the pilot program (11% from the most deprived quintile compared with 21%, respectively, and 78% White British compared with 68%, respectively).

### Comparison With Prior Work

Recent qualitative work examining how the face-to-face NHS-DPP was understood by service users reported variations in the understanding of key intervention content [26]. A study of the upstream domains of fidelity (delivery) of the face-to-face program identified underdelivery of some self-regulatory BCT content in the program [33], which may have accounted for why participants were not able to describe a clear understanding of some BCTs. Although the BCT *self-monitoring of behaviors* tended to be understood with ease, the study [26] of the face-to-face NHS-DPP found that some BCTs such as *action planning* and *problem solving* were less well understood by some recipients, unless additional support was provided. This suggests that expecting people to understand and use BCTs by themselves is unrealistic.

The findings from our study of the digital version of the program also highlight the importance of support to help participants understand and use key BCT content in the program; however, within the digital context, the nature of this *support* is different, that is, via remote health coaching rather than peer or facilitator support provided in a group setting. The factors that affect the need for support to understand BCTs have been investigated in a previous qualitative study nested within a feasibility study of a walking intervention [34]. This study investigated participants’ understanding, experiences, and enactment of a range of self-regulatory BCTs and concluded that age is an important factor that can influence how people understand and use these BCTs, with problems more common in older adults (although cognitive ability and employment status can also contribute) [34]. Participants from this study [34] and our study are of a similar age range. Accordingly, the findings from our study build on this by suggesting that providing *support* for understanding and using BCTs is a potential approach to aid their usefulness to older adults.

Furthermore, there is some commonality between evaluations of the face-to-face and digital versions of the NHS-DPP, in that *self-monitoring of behaviors* seems to be understood and used with ease. Our findings on the straightforward use of the self-monitoring and feedback BCTs via provider apps are consistent with previous work suggesting that use of digital technologies has resulted in greater ease and frequency of self-monitoring of behaviors [35]; for example, a qualitative study [36] was conducted with people at moderate-to-high risk of developing T2DM who took part in an intervention comprising a flash glucose monitor and a physical activity monitor. Individuals intuitively used, interpreted, and acted on feedback from wearable technologies, suggesting that accessing behavioral and physiological feedback can increase self-awareness of how behaviors affect short-term health. Furthermore, theme descriptions from a synthesis of qualitative studies [37] resonated with features of how self-monitoring was understood in our study, namely, an increase in self-knowledge, increased attention and adherence, and facilitation of self-analysis.

However, although variations in the understanding and use of BCTs among participants of the face-to-face NHS-DPP could be attributed to the underdelivery of self-regulatory BCTs, the same conclusion cannot be drawn for the digital version of the program. The findings to date suggest that the fidelity of behavior change content in the NHS-DDPP is better than that previously documented for the face-to-face program in terms of design [22].

Accordingly, and in line with the findings reported in this study, we suggest that *mode of delivery* of BCTs is an important factor that influences how participants understand and use key behavior change content in the NHS-DDPP. It is conceivable that *human* coaching helps service users to really understand BCTs and how to apply them to themselves. It has been proposed that *form of delivery* is a vital part of any behavior change intervention [38], in that it is an *active ingredient* in itself that serves a key function. Our study is novel in highlighting the impact of *mode of delivery* directly on the understanding and use of specific self-regulatory BCTs in a major nationally implemented behavior change program.

In more general terms, the importance of health coaching or *human support* for behavior change has been documented in previous studies. A systematic review investigating *mode of delivery* on the efficacy of internet-based interventions to promote health behavior change [39] found that the use of communicative functions to deliver BCTs via digital interventions (eg, having access to an advisor) was effective in supporting behavior change. Further studies on health coaching are consistent with our findings on the importance of its role in providing emotional support [40,41] and tailoring intervention content, including BCTs [41,42].

There is emerging literature that explores how technology could be used to provide support to people for the BCTs *problem solving* or *goal setting* without the need for *human* support. Although this is possible—for example, by digitally guiding people through a stepwise process for problem solving—most digital interventions for problem solving use generic scenarios rather than users’ own experiences or context [43]. Similarly, few technologies have so far been developed to identify and facilitate shared discussion and prioritization of meaningful, individualized goals [44]. This suggests that there are currently limits to how personalization of such BCTs can be delivered digitally, again supporting the notion that human support is central to helping users to understand and use these BCTs.

We are not aware of previous research that has directly investigated NHS-DDPP or NHS-DPP participants’ experiences of feedback on T2DM risk, although some work on communicating breast cancer risk suggests that risk feedback can help engagement with behavior change programs [45].

### Implications

Overall, this study highlights the importance of the role of health coaches in delivery of NHS-DDPP behavior change content and emphasizes how participants value coaches who provide them with emotional support and an understanding of how to use BCTs to support their behavior change journey. It is therefore recommended that future iterations of the NHS service specification spell out the need to include health coaching to support service users to understand and use key behavioral content of the program. Our findings indicate that this is most relevant to specific self-regulatory BCTs such as *feedback on behavior*, *goal setting (behaviors)*, *review behavior goals*, and *problem solving*.

The implication of our finding regarding service users’ frustration over lack of feedback on T2DM risk is an important one to consider for the NHS-DDPP. A review of the requirements of the 3 iterations of the NHS-DPP specification has shown that the requirement for providers to conduct blood tests to assess T2DM risk has changed over time; this was a clear requirement in the first version of the specification (face-to-face program only), but in the most recent version, service users are directed toward seeking a blood test from their general practitioner. Our findings indicate that this reduction in focus on feedback on T2DM risk is inconsistent with service user values and could potentially affect their motivation for maintenance of behavior change. Therefore, we suggest that the options for providing a clearer route to access such feedback via the program are explored further; this could include amending the NHS service specification for the discharge process for participants reaching the end of the program.

Our findings also have wider implications for the development and implementation of other digital behavioral interventions that deliver self-regulatory BCTs. It is encouraging that some BCTs such as *self-monitoring of behavior*, which are widely used in digital apps, seem to be understood and used by our study participants. However, the need for coaching support or human interaction to help end users understand and use other self-regulatory BCTs is an important consideration for development of future digital interventions.

More broadly, our findings suggest that *mode of delivery* of BCTs is an important factor that influences how participants understand and use key behavior change content. We cannot assume that receipt of a BCT delivered by websites, apps, and human coaches is understood and used on equal terms; this in turn is likely to affect how a person is able to enact behavior change in day-to-day life. Thus, optimizing *mode of delivery* is a priority for future research. This should include considering *mode of delivery* across a wider range of interventions and behaviors. Of course, *engagement* with any mode of delivery is a prerequisite of understanding and use; therefore, examining how service users engage with various modes of delivery, including health coaching, is an important question to be answered in future research. Further research to understand the impact of lack of feedback on T2DM risk on motivation and long-term maintenance of behavior change of NHS-DDPP participants would also be valuable.

### Conclusions

NHS-DDPP participants benefit from health coach support to understand and use some BCT content of the program, although, conversely, some BCTs delivered digitally via provider apps seem to be straightforward to understand and use. Participants expressed frustration over the lack of monitoring or feedback regarding their T2DM risk within the program. Improvements could be made to the NHS-DDPP, such as explicitly setting out the need for health coaches to support the understanding of BCT content in the service specification and amending the discharge process so that service users are able to understand any change in their T2DM risk. Developers and implementers of other digital health interventions could learn from this work by taking careful consideration of exactly when and where human support is required to help service users understand and use key behavior change content within interventions.

## Appendix

Multimedia Appendix 1 Breakdown of number of study invitations sent out by each provider.

Multimedia Appendix 2 Topic guides for interviews.

Multimedia Appendix 3 Findings related to 'action planning'.

## Abbreviations

BCT: behavior change technique
HbA1c: glycated hemoglobin
NHS: National Health Service
NHS-DDPP: National Health Service Digital Diabetes Prevention Programme
NHS-DPP: National Health Service Diabetes Prevention Programme
NIH-BCC: National Institutes of Health Behavior Change Consortium
T2DM: type 2 diabetes mellitus
