# Supplementary material for: How the Behavior Change Content of a Nationally Implemented Digital Diabetes Prevention Program Is Understood and Used by Participants: Qualitative Study of Fidelity of Receipt and Enactment
Source: J Med Internet Res. 2023 Jan 11;25:e41214. doi: 10.2196/41214 (PMC9878374; doi:10.2196/41214)
Supplement: Multimedia Appendix 1 [file jmir_v25i1e41214_app1.docx]

**Multimedia Appendix 1: Breakdown of number of study invitations sent out by each provider.**

|  | *Provider A* | *Provider B* | *Provider C* | *Provider D* |
| --- | --- | --- | --- | --- |
| *Number invitations sent in first round* | *33* | *184* | *112* | *59* |
| *Number of invitations sent in second (and third) round* |  | *38* | *7 (+17)* | *130* |
| *Total number of invitations sent* | *33* | *222* | *136* | *189* |
| *Final number of participants at timepoint 1* | *12* | *11* | *10* | *12* |
| *Final number of participants at timepoint 2* | *10* | *8* | *7* | *11* |
