# Supplementary material for: How the Behavior Change Content of a Nationally Implemented Digital Diabetes Prevention Program Is Understood and Used by Participants: Qualitative Study of Fidelity of Receipt and Enactment
Source: J Med Internet Res. 2023 Jan 11;25:e41214. doi: 10.2196/41214 (PMC9878374; doi:10.2196/41214)
Supplement: Multimedia Appendix 2 [file jmir_v25i1e41214_app2.docx]

**Multimedia Appendix 2: Topic guides for interviews**

**Interview 1: Questions about the Digital ‘Healthier You’ Diabetes Prevention Programme**

1. How were you directed towards taking part in the digital Diabetes Prevention Programme?
2. Before you started the ‘Healthier You’ digital sessions, what did you expect from the online course?
   1. Was the online course similar or different to what you were expecting?
3. What do you think the main aim of the course is?
   1. How does it try to do that?
   2. Was this made clear to you at the start of the course?
4. How often have you been accessing this online programme so far?
   1. Is this what you expected?
   2. What would encourage you to engage with and access the programme more often?
5. Can you describe the support you received when you were first enrolled onto the digital programme?
   1. What were you asked to do during your first phone call with the health coach?
      1. Introduction to digital programme?
      2. Goal setting?
      3. Tracking behaviours/weight?
   2. How did you feel after this initial phone call with your health coach?
   3. What contact have you had with the health coach since this initial phone call?
6. What information have you been given on the course so far about diabetes and preventing diabetes?
   1. Was it the right type, amount, format, level of difficulty?
   2. Is there any information that has encouraged you to change aspects of your current lifestyle?
7. What programme activities have you been asked to compete so far?
   1. Reading?
   2. Interactive content (e.g. videos, worksheets, external websites)?
   3. Interaction with health coach?
   4. Interaction with others also taking the course?

**(Elicit description of components, then in turn):**

- - **Have you completed this?**
  - **What do you think this activity was trying to achieve?**
  - **How useful did you find this for helping to change your diet and physical activity?**

1. What support have you received on the programme so far?
   1. One-to-one coaching?
   2. Group support from others on the programme?
   3. Support from family and friends?
   4. External websites?

**(Elicit description of components, then in turn):**

- - **How have you found this support?**
  - **How useful did you find this support for helping to change your diet and physical activity?**

1. So far, have you been asked to set any goals or targets for your physical activity, or diet, or weight loss?
   1. How are you getting on with that? (Choosing, setting, working towards goal?)
   2. How do you think goals might *work* to help people change their physical activity or diet?
   3. How *useful* are you finding this for changing your physical activity or diet?
2. Have you been asked to make a more detailed plan for changing your diet or physical activity, sometimes called an action plan?
   1. How are you getting on with that? (Making a plan, sticking to it?)
   2. How do you think action plans might *work* to help people change their physical activity or diet?
   3. How *useful* are you finding this for changing your physical activity or diet?
3. Have you been asked to keep track of your physical activity or diet or weight, by using a daily diary/log or a step counter?
   1. How are you getting on with that?
   2. How do you think keeping track of things in this way might *work* to help people change their physical activity or diet?
   3. How *useful* are you finding this for changing your physical activity or diet?
4. Have you been asked to think about any barriers that get in the way of making healthy changes and come up with solutions to getting over these barriers?
   1. How did you get on with that?
   2. How do you think problem solving in this way might *work* to help people change their physical activity or diet?
   3. How *useful* did you find this for changing your physical activity or diet?
5. Have you been given any feedback so far on changes to your weight, blood glucose levels, diet or physical activity?
   1. How *useful* did you find it to have that feedback?
   2. How do you think getting feedback in this way might *work* to help people change their physical activity or diet?
   3. How did you feel when you were given that feedback?
   4. How timely was the feedback?
6. Has there been anything else on the course so far that you have found useful?
7. Is there anything else about the course we haven’t talked about yet that you’d like to tell me about or report back on?

*Thank you very much for taking the time to take part in our research.*

**Interview 2: Questions about the Digital ‘Healthier You’ Diabetes Prevention Programme**

[Note questions and prompts tailored to individual, based on responses in first interview]

1. Since we last spoke, how often have you accessed this online programme?
   1. Is this what you expected?
   2. What would encourage you to engage with and access the programme more often?
2. Since, we last spoke, have there been any changes to the programme that you have received? If so, how have you found them?
3. Have you been given any more information on the course since we last spoke about diabetes and preventing diabetes?
   1. How have you found the information that you have received on the programme (e.g. type, amount, level of difficulty)?
   2. Is there any information that you particularly remember and will take away with you?
4. What activities have you been asked to complete since we last spoke?
   1. Reading?
   2. Interactive content (e.g. videos, worksheets, external websites)?
   3. Interaction with health coach?
   4. Interaction with others also taking the course?

**(Elicit description of components, then in turn):**

- - **Have you completed this?**
  - **What do you think this activity was trying to achieve?**
  - **How useful did you find this for helping to change your diet and physical activity?**

1. Since we last spoke, what support have you received on the programme?
   1. One-to-one coaching?
      1. What has your relationship been like with your health coach?
      2. How often have you communicated with your health coach?
      3. How helpful have you found this one-to-one coaching?
      4. How important was your health coach for sticking to the online programme and making changes to your lifestyle?
   2. Group support from others on the programme?
      1. How important were these group forums for helping you to make changes to your lifestyle?
   3. Support from family and friends?
      1. How important were your family and friends for sticking to the online programme and making changes to your lifestyle?
      2. What impact did their views and behaviours have?
   4. External websites?

**(Elicit description of components, then in turn):**

- - **How have you found this support?**
  - **How useful did you find this support for helping to change your diet and physical activity?**
  - **Did you feel the support was tailored to your needs? If so, how?**

1. Since we last spoke, have you been asked to set any more goals or targets for your physical activity, or diet, or weight loss?
   1. How are you getting on with that? (Choosing, setting, working towards goal?)
   2. How do you think goals might *work* to help people change their physical activity or diet?
   3. How *useful* are you finding this for changing your physical activity or diet?
2. Since we last spoke, have you been asked to make a more detailed plan for changing your diet or physical activity, sometimes called an action plan?
   1. How are you getting on with that? (Making a plan, sticking to it?)
   2. How do you think action plans might *work* to help people change their physical activity or diet?
   3. How *useful* are you finding this for changing your physical activity or diet?
3. Since we last spoke, have you been asked to keep track of your physical activity or diet or weight, by using a daily diary/log or a step counter?
   1. How are you getting on with that?
   2. How do you think keeping track of things in this way might *work* to help people change their physical activity or diet?
   3. How *useful* are you finding this for changing your physical activity or diet?
4. Since we last spoke, have you been asked to think about any barriers that get in the way of making healthy changes and come up with solutions to getting over these barriers?
   1. How did you get on with that?
   2. How do you think problem solving in this way might *work* to help people change their physical activity or diet?
   3. How *useful* did you find this for changing your physical activity or diet?
5. What feedback have you been given on changes to your weight, blood glucose levels, diet or physical activity?
   1. How *useful* did you find that feedback?
   2. How do you think getting feedback in this way might *work* to help people change their physical activity or diet?
   3. How did you feel when you were given that feedback?
   4. How timely was the feedback?
6. Have you had your final discharge call with your health coach yet?
   1. What did you discuss in this phone call?
      1. Feedback on progress?
      2. Goals?
      3. Signposting?
   2. How useful did you find this phone call?
   3. **If not had the call yet – what would you expect/like to have at the end of the programme in terms of support?**
7. What do you think of digital programme, now that you’ve used it?
   1. What did you like about it?
   2. What did you not like?
   3. What was important for you?
   4. How could it be improved?
8. Overall, how useful did you find the digital Diabetes Prevention Programme?
   1. What is the main thing in your life that changed for you as a result of this intervention?
   2. Did your use change over time?
      1. What did you use more/ less over time? Why?
   3. Was there anything that you would have liked to have seen included?
   4. What did you think of:
      1. Content
      2. Presentation
      3. Tone
      4. Connection with web links, email, text, etc.
      5. Forum, videos, local resources
9. Now that you are near/at the end of the course, what do you think the main aim of the course was?
   1. How did it try to do that?
10. Is there anything else about the overall course we haven’t talked about yet that you’d like to tell me about or report back on?

*Thank you very much for taking the time to take part in our research. This is our final interview with you.*
