# Supplementary material for: How the Behavior Change Content of a Nationally Implemented Digital Diabetes Prevention Program Is Understood and Used by Participants: Qualitative Study of Fidelity of Receipt and Enactment
Source: J Med Internet Res. 2023 Jan 11;25:e41214. doi: 10.2196/41214 (PMC9878374; doi:10.2196/41214)
Supplement: Multimedia Appendix 3 [file jmir_v25i1e41214_app3.docx]

**Multimedia Appendix 3** Findings related to 'action planning'**.**

**Scant delivery and openness to ‘action planning’**

Few participants recalled or accessed the BCT ‘action planning’ within the programme (with the exception of Provider C). Even for participants taking part in the Provider C programme, there was still some variation in how participants understood this BCT, ranging from seeing ‘action planning’ as the detail of ‘how’ to reach goals and occasional reference to support from the Health Coach to help them do this, to others that appeared to be using this technique in a self-directed way. Across all providers there was notable scepticism about the use of ‘action planning’. Participants preferred to ‘just do it’ implying that they view this technique as an unnecessary step in behaviour change, or perhaps see ‘action planning’ as too ‘business-like’ based on their experiences from their work life.

*“But I just - an action plan for me is a reminder that you need to do something within a certain time period and provides a robust framework to work to. I don’t think that’s required on this. If you’re exercising and you’re eating sensibly at the times and eating the right foods, I don’t need to write it down nor do I need reminding that I need to do it (Male, 57 years, Provider D)”*
